# Optical coherence tomography (OCT) and OCT-angiography in syndromic versus non-syndromic *USH2A*-associated retinopathy

**DOI:** 10.1177/11206721241247421

**Published:** 2024-04-11

**Authors:** Alessio Antropoli, Alessandro Arrigo, Carlo Caprara, Lorenzo Bianco, Stefano Mercuri, Alessandro Berni, Ilaria Passerini, Sofia Gambarotta, Andrea Sodi, Francesco Bandello, Vittoria Murro, Maurizio Battaglia Parodi

**Affiliations:** 1 Department of Ophthalmology, IRCCS San Raffaele Scientific Institute, Milan, Italy; 2 18985 Vita-Salute San Raffaele University, Milan, Italy; 3 Department of Neurosciences, Psychology, Drug Research, and Child Health, Eye Clinic, University of Florence, AOU Careggi, Florence, Italy

**Keywords:** Usher syndrome, non-syndromic retinitis pigmentosa, multimodal imaging, OCT, OCTA

## Abstract

**Purpose:**

To compare non-syndromic and syndromic forms of *USH2A*-related retinitis pigmentosa (RP) by means of structural optical coherence tomography (OCT) and OCT-angiography (OCTA).

**Methods:**

Observational, cross-sectional, multicenter study. All patients underwent best corrected visual acuity (BCVA) measurement, OCT (Spectralis HRA + OCT, Heidelberg Engineering) and OCTA (OCT DRI Topcon Triton, Topcon Corporation). We compared subfoveal choroidal thickness (SCT), choroidal vascularity index (CVI), presence of cystroid macular edema (CME), macular vessel density (VD) at the superficial and deep capillary plexa, as well as VD of the radial peripapillary capillary (RPC) network, between syndromic and non-syndromic patients with *USH2A*-associated retinopathy.

**Results:**

Thirty-four eyes from 18 patients (7 females) were included. Thirteen patients (72.2%) were affected by Usher syndrome type 2, whereas the remaining 5 subjects (27.8%) had non-syndromic retinitis pigmentosa (nsRP). Syndromic patients were younger than nsRP (*p* = 0.01) and had a worse visual acuity than those with the exclusively retinal phenotype. Patients with Usher syndrome type 2 had a higher prevalence of CME and a thicker choroid compared to nsRP, although these results were not statistically significant (*p* = 0.775 and *p* = 0.122, respectively). Similarly, none of the other quantitative OCT and OCTA parameters was statistically different between the two groups.

**Conclusions:**

Despite their younger age, patients with Usher syndrome type 2 displayed similar choroidal and microvascular changes compared to those with nsRP.

## Introduction

Retinitis pigmentosa (RP) includes a group of inherited retinal diseases characterized by progressive degeneration of the rod and cone photoreceptors, which results in visual loss.^1–6^ Mutations in genes involved in the ciliary function can result in syndromic forms of RP. Among these, the most common is Usher Syndrome type 2, caused by biallelic pathogenic variants in the *USH2A* gene and recognized as the most frequent cause of deaf-blindness in humans.^7,8^

In addition to the retinal degeneration, many optical coherence tomography (OCT) and OCT-angiography (OCTA) studies have described the presence of vascular defects in the superficial and deep capillary plexa, as well as a reduction in subfoveal choroidal thickness (SCT) in patients affected by RP.^9–15^ Nonetheless, only few studies directly compared the structural and vascular impairment between syndromic and non-syndromic patients with *USH2A*-related RP. Studies available on this topic mainly focused on genotype-phenotype correlations or focalized on the prevalence of comorbidities (such as macular hole and epiretinal membrane) or structural differences on OCT images.^16–18^

In addition, OCTA data specifically pertaining patients with Usher syndrome have been relatively understudied, with no studies directly comparing microvascular alterations among syndromic and non-syndromic forms of *USH2A*-related RP.^19^

Therefore, we designed this study with the primary aim of comparing structural, microvascular and choroidal changes in syndromic versus non-syndromic *USH2A*-related retinopathy.

## Materials and methods

This cross-sectional, multicenter study was part of a collaborative project (NET-2016-02363765) focused on phenotyping and genotyping invidividuals with RP. Patients with a molecularly confirmed diagnosis of *USH2A*-associated retinopathy were retrospectively identified from the institutional genetic databases of the Italian Ophthalmology Departments of IRCCS San Raffaele (Milan) and Hospital and Eye Clinic, Careggi Teaching Hospital (Florence), which served as the participating sites for the study. Based on the patients’ past medical records (e.g., cochlear implantation, sensorial hearing loss testified by audiometry exam) we classified subjects into syndromic and non-syndromic RP.^8,20^

The ophthalmological examinations consisted of best corrected visual acuity (BCVA) measurement using Early Treatment Diabetic Retinopathy Study (ETDRS) charts, optical coherence tomography (OCT) and OCTA scans. Structural OCT images were acquired with Spectralis HRA + OCT (Heidelberg Engineering, Heidelberg, Germany). OCTA images were obtained using a swept-source OCT DRI Topcon Triton (Topcon Corporation, Tokyo, Japan) device. The research followed the tenets of the Declaration of Helsinki and was approved by the local institutional review board (study ID: MIRD). Written informed consent was obtained from all recruited subjects.

### Image analysis

The OCT acquisition protocol consisted of a 20° × 15° (5.6 × 4.2 mm) raster volume scan centered on the fovea and composed of 19 horizontal B-scans (235 µm interscan distance) with an average of 9 Automatic Real-time Tracking (ART) frames each.^21^ Each image was reviewed for the presence of cystoid macular edema (CME). Horizontal 30° OCT scans encompassing the fovea were used to calculate the SCT and the choroidal vascularity index (CVI). The distance between the Bruch's membrane and the sclerochoroidal interface in the subfoveal location was manually outlined by a single trained grader (AA), as this method has demonstrated substantial repeatability.^22,23^ CVI was calculated as already reported in literature.^24^ In short, using the ImageJ software, we selected the choroid as the region of interest. After binarization, the CVI was calculated by determining the ratio of the luminal choroidal area (black pixels) to the total choroidal area (black + white pixels).

The superficial capillary plexus (SCP) and deep capillary plexus (DCP) in the macula, and the radial peripapillary capillary network (RPC) at the optic nerve head were automatically segmented on en-face OCTA 4.5 × 4.5 mm images. Each reconstruction was reviewed by an expert ophthalmologist (AA) and manually corrected, if necessary. All reconstructions were exported in .tiff format and loaded into the ImageJ software for calculating vessel density (VD), as already described elsewhere.^25^ For the RPC, VD was calculated before and after skeletonization (sRPC) to reduce the impact of the great vessels on our measurements.

### Statistical analysis

Analyses were performed using R studio (version 2023.03.0 + 386). All descriptive data were expressed as mean ± standard deviation (SD) for continuous normally distributed variables, as median (interquartile range, IQR) for non-normal ones, and as frequency and percentages for categorical ones. Testing for normality of continuous variables was assessed using the Shapiro-Wilk test. Correlations between BCVA and OCTA quantitative metrics were performed for the right eyes with the Spearman's test. The Bonferroni correction was subsequently applied to account for the risk of type I errors due to multiple comparisons. Comparisons between groups were performed with either a two-sided Student's t-test, linear mixed models (LMM) or generalized linear mixed models (GLMM), as appropriate. Inter-eye correlations were accounted by setting the “subject” variable as a random effect, while patients’ age and phenotype were treated as fixed factors.

## Results

A total of 34 eyes from 18 patients (7 females) diagnosed with RP due to pathogenic variants in the *USH2A* gene were included. Two eyes from 2 patients were discarded because of poor image quality. Thirteen (72.2%) patients were affected by Usher syndrome type 2, whereas the remaining 5 (27.8%) subjects solely manifested the retinal phenotype. Two patients harbored previously unpublished variants. One individual was homozygous for the c.13130C>T p.Ser4377Leu missense mutation, while another patient exhibited the same variant in compound heterozygosity with the c.12712T>C p.Tyr4238His. Notably, an additional patient carried a large intragenic deletion spanning from exon 45 to exon 47 in a compound heterozygous state. Furthermore, for two patients only one pathogenic variant could be identified. Nevertheless, due to their syndromic presentation, consistent retinal phenotype, and the absence of pathogenic variants in other genes associated with inherited retinal diseases, they were still included in this study. The genetic characteristics of our cohort are outlined in Table 1.

**Table 1.** List of USH2A variants in our cohort.

		Variant 1				Variant 2			
Subject	Status	DNA change	Protein change	Exon	ACMG	cDNA variant	Protein variant	Exon	ACMG
nsRP-01	HET	c.2276G > T	p.Cys759Phe	13	Class 5	c.1841-2A > G	p.?	11	Class 5
nsRP-02	HET	c.2710_2720dup	p.Leu908Profs*63	13	Class 5	c.2296T > C	p.Cys766Arg	13	Class 5
nsRP-03	HET	c.3332T > G	p.Leu1111*	17	Class 5	c.10437G > T	p.Trp3479Cys	53	Class 4
nsRP-04	HET	c.653T > A	p.Val218Glu	4	Class 5	c.2276G > T	p.Cys759Phe	13	Class 5
nsRP-05	HET	c.2276G > T	p.Cys759Phe	13	Class 5	c.10712C > T	p.Thr3571Met	54	Class 5
Ush-01	HET	c.11235C > A	p.Tyr3745*	58	Class 5	c.8655_8681 + 1681del	p.?	43	Class 5
Ush-02	HET	c.13392G > A	p.Trp4464*	63	Class 5	c.9424G > T	p.Gly3142*	48	Class 5
Ush-03	HET	c.15020C > T	p.Pro5007Leu	69	Class 5	c.2071T > C	p.Cys691Arg	12	Class 5
Ush-04	HET	c.1841-2A > G	p.?	11	Class 5	c.2299del	p.Glu767Serfs*21	13	Class 5
Ush-05	HOM	c.2276G > T	p.Cys759Phe	13	Class 5	c.2276G > T	p.Cys759Phe	13	Class 5
Ush-06	HOM	c.2299del	p.Glu767Serfs*21	13	Class 5	c.2299del	p.Glu767Serfs*21	13	Class 5
Ush-07	HET	c.2299del	p.Glu767Serfs*21	13	Class 5	c.9676C > T	p.Arg3226*	49	Class 5
Ush-08	*Unsolved*	c.7932G > A	p.Trp2644*	41	Class 5				
Ush-09	HET	c.802G > A	p.Gly268Arg	5	Class 5	c.12712T > Ca	p.Tyr4238His	63	Class 4
Ush-10	HET	c.1841-2A > G	p.?	11	Class 5	c.8628G > A	p.Trp2876Ter	43	Class 5
Ush-11	HOM	c.13130C > Ta	p.Ser4377Leu	63	Class 3	c.13130C > Ta	p.Ser4377Leu	63	Class 3
Ush-12	*Unsolved*	c.2209C > T	p.Arg737*	13	Class 5				
Ush-13	HET	c.13130C > A	p.Ser4377*	63	Class 5	Exon 45–47 Deletionb			Class 5

ACMG = American College of Medical Genetics and Genomics; nsRP = Non-syndromic Retinitis pigmentosa; Ush = Usher syndrome type 2; HET = Compound heterozygous; HOM = Homozygous.

^a^ Previously unpublished variants.

^b^ bGenomic coordinates: NC_000001.10:g.(216040427_216019204)_(216011389_215990406).

In our cohort, patients with non-syndromic retinitis pigmentosa (nsRP) were older on average than those with the syndromic form, with ages of 43 years (IQ range: 43–51) and 33 years (IQ range: 24–41), respectively (*p* = 0.01). The overall median BCVA was 0.1 logMAR (IQR 0.0–0.2), with 8 eyes (80%) from the nsRP group and 6 eyes (23.1%) from the syndromic group exhibiting a BCVA of 20/20 Snellen. Patients’ demographics and visual acuity characteristics are listed in Table 2.

**Table 2.** Patients’ demographics and visual acuity characteristics.

	Non-syndromic	Syndromic
Total eyes, n (%)	10 (29.4)	24 (70.6)
Total patients, n (%)	5 (27.8)	13 (72.2)
Age, median (IQR) (years)	43 (43–51)	33 (24–41)
Sex, n (%) (females)	2 (40)	5 (38.5)
BCVA, median (IQR) (logMAR)	0.0 (0.0–0.0)	0.2 (0.1–0.2)

IQR = Interquartile range; BCVA = Best-corrected visual acuity.

### Imaging parameters

In our cohort, CME was present in 2 (20%) out of 10 non-syndromic eyes and in 11 (41.6%) out of 24 syndromic eyes, although this difference was not statistically significant (*p* = 0.775).

Among the quantitative OCT variables analyzed, we noted a remarkable difference in mean SCT, which was 182.4 ± 75.0 µm in the nsRP group and 298.5 ± 94.2 µm in syndromic patients. By contrast, CVI was comparable, being 66.5 ± 6.1% in the nsRP group and 67.7 ± 3.6% in the Usher syndrome group. However, after accounting for age, none of the analyzed variables displayed a statistically significant difference between the two groups (*p* = 0.122 and *p* = 0.705, respectively). Moving to the OCTA parameters, our analyses unveiled a significant correlation between BCVA and the VD of the DCP, which remained significant even after applying the Bonferroni correction for multiple comparisons (Table 3).

**Table 3.** Correlation between BCVA and OCTA parameters.

Vessel densities	*Rho Spearman*	*p value*	*Bonferroni corr. p.*
Superficial capillary plexus	−0.48	0.09	0.36
Deep capillary plexus	−0.87	0.0001138	0.000455
RPC	−0.30	0.348	1
Skeletonized RPC	−0.28	0.3795	1

BCVA = Best-corrected visual acuity; RPC = Radial peripapillary capillary plexus.

Similarly, the patients’ phenotype had no effect on any of the quantitative OCTA metrics analyzed. All data are reported Table 4.

**Table 4.** Effect of phenotype on quantitative OCT and OCTA variables.

Dependent variables	Fixed effect (95% CI)^a^	p*
*OCT parameters*		
Subfoveal choroidal thickness, µm	85.4 (−12.7–183.5)	0.122
Choroidal vascularity index	−1.0 (−5.9–3.9)	0.705
*Vessel densities* ^b^		
Superficial capillary plexus, %	−1.1 (−3.9–1.7)	0.480
Deep capillary plexus, %	−2.6 (−7.5–2.2)	0.320
RPC, %	−2.1 (−7.4–3.2)	0.471
Skeletonized RPC, %	0.01 (−1.0–1.1)	0.986

OCT = Optical coherence tomography; OCTA = Optical Coherence Tomography Angiography; RPC = Radial peripapillary capillary network.

*p value for the linear mixed model regression.

^a^ Usher syndrome type 2 versus non-syndromic retinitis pigmentosa [reference status].

^b^ Expressed as percentage of white pixels. Age was set as fixed effect (data not shown).

## Discussion

RP can manifest as a syndromic phenotype, especially when mutations in genes encoding ciliary proteins occur, as happens in Usher syndrome.^8^ OCT and OCTA have been widely used in recent years to characterize RP through structural and vascular changes.^10,11,26–32^ On the other hand, remarkably few studies have focused on OCTA in Usher syndrome type 2 and, more specifically, studies comparing syndromic versus non-syndromic patients are even scarcer.^17–19,33–37^ We therefore designed this cross-sectional study with the goal of comparing syndromic and non-syndromic forms of *USH2A*-related retinopathy to verify whether the patient's phenotype could affect the severity of anatomical modifications and microvascular impairment.

In our cohort, patients with syndromic *USH2A*-related retinopathy were significantly younger than those with the exclusively retinal phenotype. The former presented with lower BCVA, even though both groups demonstrated a similar degree of vascular impairment. Nonetheless, consistent with findings from previous research, we observed a statistically significant negative correlation between BCVA and the VD of the DCP.^38^ The latter has also been shown to closely correlate with the status of the ellipsoid zone (EZ).^39^ Given that a shorter EZ is associated with poorer visual acuity,^40^ our findings could indirectly reflect these structural OCT changes, even though they were not directly assessed in this study.

In a study by Sandberg et al., they observed a similar rate of progression in syndromic and non-syndromic patients.^37^ However, patients with Usher syndrome are known to experience an earlier onset of the disease, which results in an earlier severe visual impairment and could possibly explain the disparity in BCVA that we observed in our cohort.^36^ Another possible explanation was the different prevalence of macular edema, which was 45.8% for the Usher syndrome group and 28.6% in the nsRP group. Although not statistically significant, these results are similar to the findings observed by Colombo L. et al, which highlighted a greater prevalence of cystoid maculopathy in syndromic patients.^17^ However, a closer look at our data revealed similar visual acuities between patients with and without macular edema (data not shown). Consistent with existing literature, the mere presence of CME cannot reliably predict visual acuity in RP. Instead, central macular thickness has been demonstrated to correlate more closely with visual acuity decline, warranting further investigation.^41–44^

Moving to the choroid, with OCT we studied both SCT and CVI. After accounting for patients’ age, which has been shown to correlate with SCT, we did not find any statistically significant difference between the two groups. A previous analysis by Colombo L. et al did not find any difference in SCT of nsRP and Usher syndrome patients, although their conclusions were drawn by comparing their data with previous literature.^16^ Our data confirm their results and expand this findings to CVI, which is currently regarded as a more reliable tool given the lack of influence of age on this parameter.^45,46^

To the best of our knowledge, this is the first time OCTA has been used to directly compare the choroidal and microvascular impairment in syndromic and non-syndromic forms of *USH2A*-associated retinopathy. In a previous study, Hagag AM et al. compared syndromic patients with biallelic variants in USH2A and MYO7A by means of OCT, OCTA and microperimetry. They found similar VD values for the DCP and SCP between the two groups, while a more severe impairment in CC was evident in the MYO7A group, although this last parameter was only assessed qualitatively.^19^ Our data confirm previous findings of a vascular impairment in patients affected by RP, suggesting a similar involvement of the vascular plexa in nsRP and Usher syndrome due to *USH2A*. The damage at this level in RP patients likely represents a secondary involvement of the capillary networks due to a reduced trophic request, resulting from the retinal pigment epithelium-photoreceptor complex loss.^10^

We are aware that our study is burdened by several limitations, including the study's very design as a cross-sectional investigation and the relatively small sample.

In conclusion, we compared syndromic and non-syndromic patients affected by *USH2A*-related retinopathy with OCT and OCTA, not finding significant differences in terms of vascular changes. Further larger longitudinal studies are warranted to confirm the absence of differences between nsRP and patients with Usher syndrome type 2 and to investigate the influence of specific mutations on these patients’ anatomical and functional parameters.
